# New solution for fast axial scanning in fluorescence microscopy

**DOI:** 10.1038/s41377-020-00442-0

**Published:** 2020-12-14

**Authors:** Weijian Zong

**Affiliations:** Kavli Institute for Systems Neuroscience and Centre for Neural Computation, Norwegian University of Science and Technology, Trondheim, Norway

**Keywords:** Microscopy, Light-sheet microscopy

## Abstract

A novel technique based on the remote-focusing concept, using a galvanometer scanner combined with a self-fabricated “step mirror” or “tilted mirror” to transform fast lateral scanning into axial scanning, was reported as a new solution for fast, subcellular, 3D fluorescence imaging.

Fluorescence imaging techniques, such as multiphoton microscopy (MPM) and light-sheet fluorescence microscopy (LSFM), have led to rapid advances in cell biology, developmental biology, neuroscience and many other fields. Thanks to thin optical sectioning, high spatial and temporal resolution, and deep penetration into scattering tissues, these imaging technologies have allowed an unprecedented look into many details of subcellular structure and dynamics in living animals^1^. The focus of many studies has recently shifted from the single-cell level to the populational level and from two dimensions (2D) to three dimensions (3D). However, the side effects of thin optical sectioning are limited cell yield if imaging is performed in only a single plane. Fast axial scanning is therefore highly desirable. In recent years, many novel techniques have been presented to approach this goal. For example, adding an electrical tunable lens (ETL) in the conjugated pupil plane has become a popular way to perform rapid Z-scanning^2,3^, and ETLs have been integrated into several commercial systems. Remote focusing by introducing a module with another objective and piston-moving mirror is another elegant method with the additional benefit of aberration compensation^4^. Spatial/temporal multiplexing is another recent rapid z-scanning technology that has extended the axial scanning speed beyond the kHz range in a semisynchronous fashion^5–7^ (Fig. 1).

**Fig. 1:**
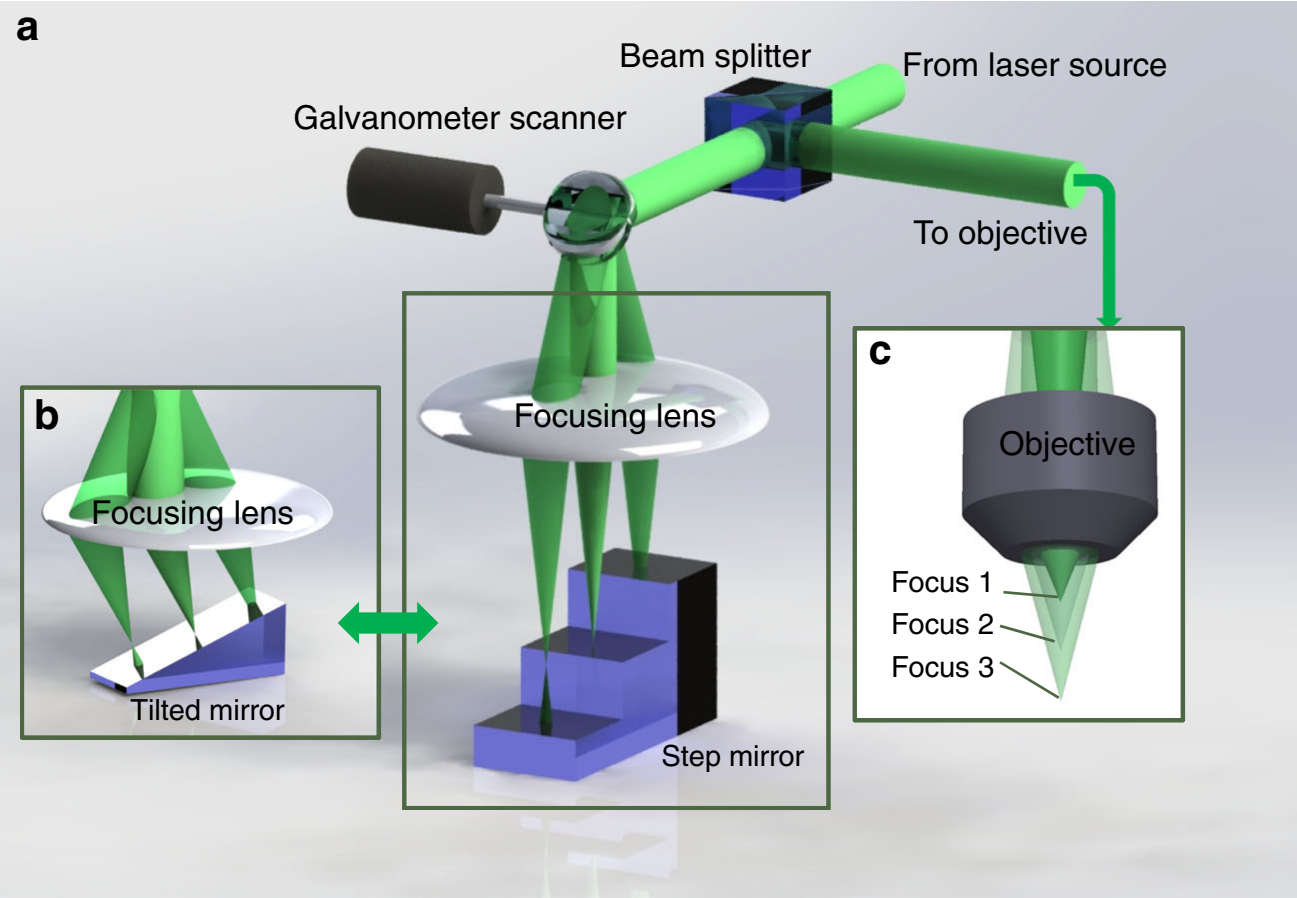
The simplified concept of the technique reported in ref. ^8^. **a** Different focal planes were selected by lateral scanning of the laser with the galvanometer scanner and focusing lens at different depths of the “step mirror”. **b** Continuous focus changing was achieved by replacing the “step mirror” with the “tilted mirror”. **c** The lateral scanning in (**a**) and (**b**) was then transformed to axial scanning under the imaging objective.

To address this grand challenge, Fiolka et al. provide a novel solution based on remote focusing^8^. Instead of using a piston actuator to drive the movement of the mirror forward and backward, they use a galvanometer scanner combined with a self-fabricated “step mirror” or “tilted mirror” to transform fast lateral scanning into axial scanning. Compared to the previous approaches, the new method has several advantages. First, the lateral scanning device, such as the galvanometer scanner, can achieve speeds ~one order of magnitude higher than the ETL. Thanks to the well-established controlling hardware, the galvanometer also features a more linear response and more reliable performance. Second, more galvanometer options are available from various companies than piston-moving mirror module options. Third, the axial scanning range can be highly customized by designing and fabricating different “step/tilted mirrors”. An especially tempting prospect of this technology is that in principle, it could be combined with other techniques, such as spatial/temporal multiplexing or holographic pattern generation.

With the impressive results that the authors showed here, we believe that this technique may be very useful in many applications, such as the study of intracellular 3D dynamics, multilayer neuron network recording, and functional imaging of fast-moving organisms, such as zebrafish embryos, *C. elegans*, or fly larvae. At the same time, however, we also envision some open questions and potential for further development of this technique. For example, can step/tilted mirrors be easily fabricated at a reasonable cost so that this technique can be widely applied? Can this technique be conveniently adapted to upgrade existing custom or commercial systems? For future developments, an interchangeable mechanical design allowing users to choose freely among multiple step/tilted mirrors with different scanning ranges may be interesting.
